# Endoscopic Overlay Myringoplasty: A Feasible Endoscopic Variant With Potential for Improved Graft Stability

**DOI:** 10.7759/cureus.94613

**Published:** 2025-10-15

**Authors:** Ismail Nakkabi

**Affiliations:** 1 Ear, Nose, and Throat (ENT) Department, Hôpital Militaire Oued Eddahab, Agadir, MAR

**Keywords:** cartilage graft, endoscopic myringoplasty, graft stability, overlay technique, tympanic membrane perforation

## Abstract

Overlay myringoplasty is a well-established technique for repairing challenging tympanic membrane perforations, particularly anterior and subtotal defects. We report our experience with an endoscopic variant using cartilage grafts, which appears feasible and may offer favorable graft stability. This retrospective case series included five patients who underwent endoscopic cartilage overlay myringoplasty between February 2024 and February 2025. The grafts were placed laterally to the fibrous layer and medially to the tympanic membrane skin and canal skin flap. Anatomical and functional outcomes were evaluated postoperatively. Successful anatomical closure was achieved in all cases, with measurable hearing improvement. No complications such as graft lateralization, blunting, infection, or epithelial cyst formation were observed. The endoscopic approach facilitated visualization of the anterior margin and allowed precise graft placement. Endoscopic overlay myringoplasty represents a reliable and feasible technical variant. By combining the advantages of the overlay principle with endoscopic exposure, this approach may provide favorable graft stability and satisfactory functional outcomes, especially in challenging anterior or subtotal perforations.

## Introduction

Tympanic membrane perforation is a common condition in otologic practice, often resulting from chronic otitis media, trauma, or iatrogenic causes. Myringoplasty remains the standard surgical procedure to restore tympanic membrane integrity, improve hearing, and prevent recurrent infections [1].

Several techniques have been described, with the underlay and overlay approaches being the most widely used. The underlay technique is simple and effective in most cases, but anterior and subtotal perforations remain challenging due to limited visualization and an increased risk of anterior graft failure [2]. The overlay technique offers better exposure and reliable closure of such perforations but has historically been associated with longer healing times and potential complications, including lateralization or blunting [3].

The advent of endoscopic ear surgery has significantly enhanced visualization of the tympanic membrane, particularly its anterior portion, and has reduced the need for canalplasty [4]. This has renewed interest in modified overlay techniques that may combine the advantages of wide exposure with minimally invasive access [2]. Furthermore, the use of cartilage grafts has gained popularity, as they provide better mechanical stability and resistance to resorption compared to temporalis fascia, while maintaining favorable hearing outcomes [1,5,6].

In this context, we report a small series of patients who underwent an endoscopic overlay myringoplasty with preservation of the fibrous layer, a technical variant designed to enhance graft stability while minimizing complications.

## Case presentation

We report a series of five patients (three females and two males; age range 18-45 years; mean age 29 years) who underwent endoscopic overlay myringoplasty with preservation of the fibrous layer between February 2024 and February 2025 at our tertiary referral center. All patients presented with large central or anterior tympanic membrane perforations and had dry ears at the time of surgery, without evidence of cholesteatoma, active infection, or ossicular chain disruption.

The diagnosis of cholesteatoma was excluded based on clinical and otoscopic findings. All ears were dry, with no epidermal debris or cholesteatomatous pearls suggesting cholesteatoma or other pathology requiring imaging. Furthermore, the preoperative air-bone gap (ABG) did not exceed 30 dB in any case, suggesting an intact ossicular chain in our practice. Therefore, the hearing loss was considered to be related to the tympanic membrane perforation rather than ossicular disruption.

All procedures were performed under general anesthesia with endotracheal intubation. Patients were positioned supine with the head slightly elevated and rotated contralaterally. A 30° rigid endoscope (4 mm, 18 cm; Karl Storz, Tuttlingen, Germany) connected to a 4K high-definition video system was used in all cases. The endoscope was held in the surgeon's left hand, leaving the right hand free for instrumentation. Dedicated otologic instruments from the Panneti endoscopic ear surgery set, designed with integrated suction capability, were employed, and suction intensity was controlled using a Portmann foot pedal.

The surgical steps

Perforation Assessment

**Figure 1:**
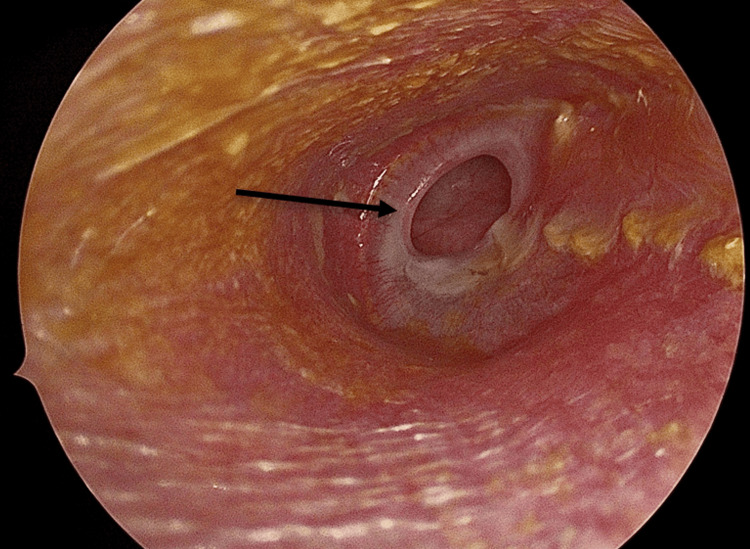
Perforation evaluation (black arrow)

The tympanic membrane perforation was carefully inspected, and the perforation margins were refreshed with a sickle knife (Figure 1).

Tympanomeatal Flap Elevation

**Figure 2:**
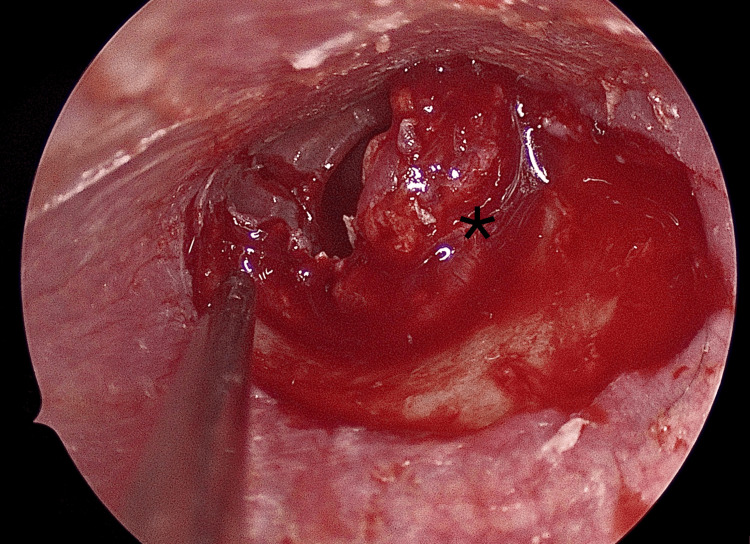
Tympanomeatal flap elevation (asterisk)

Two counter-incisions were made using Plester knives. A Rosen knife was then used to perform a classical tympanomeatal incision from 12 o'clock to 6 o'clock, approximately 2 mm lateral to the annulus. A tympanomeatal flap was elevated in the usual fashion, but the annulus itself was not elevated (Figure 2).

Dissection Over the Fibrous Layer

**Figure 3:**
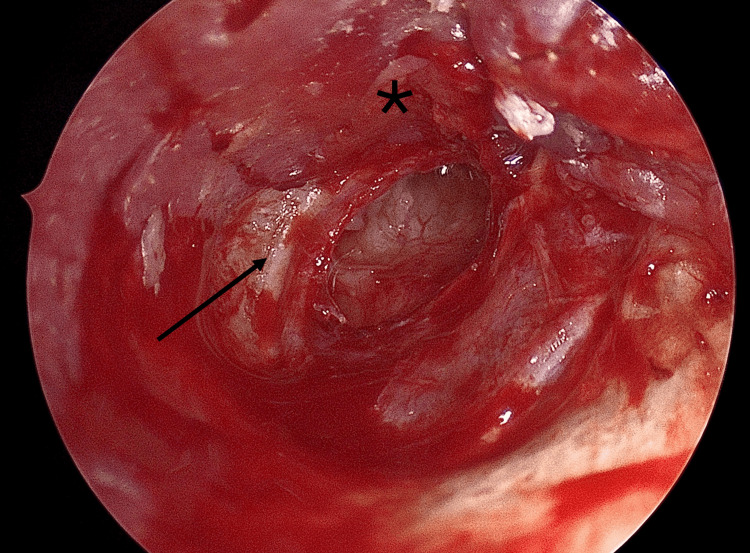
Dissection of the skin (asterisk) over the fibrous layer (black arrow)

At the annular level, dissection was continued by separating the cutaneous layer of the tympanic membrane from its fibrous layer, while preserving the latter. This step created a potential plane for graft placement while maintaining structural support (Figure 3).

Cartilage Graft Harvesting and Preparation

Tragal cartilage was harvested through a small incision, preserving perichondrium on the lateral surface. The graft was thinned to approximately 0.5 mm and trimmed to overlap the perforation margins by 1-2 mm.

Graft Placement (Overlay Principle)

**Figure 4:**
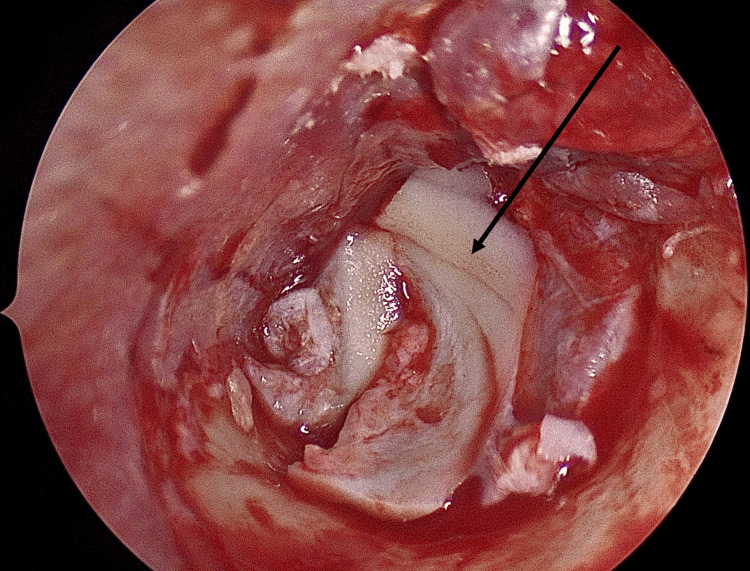
Graft (black arrow) placement

The prepared cartilage graft was positioned laterally to the preserved fibrous layer and medially to the elevated cutaneous flap, ensuring stable closure of the perforation and reliable anterior support (Figure 4).

Flap Repositioning and Stabilization

**Figure 5:**
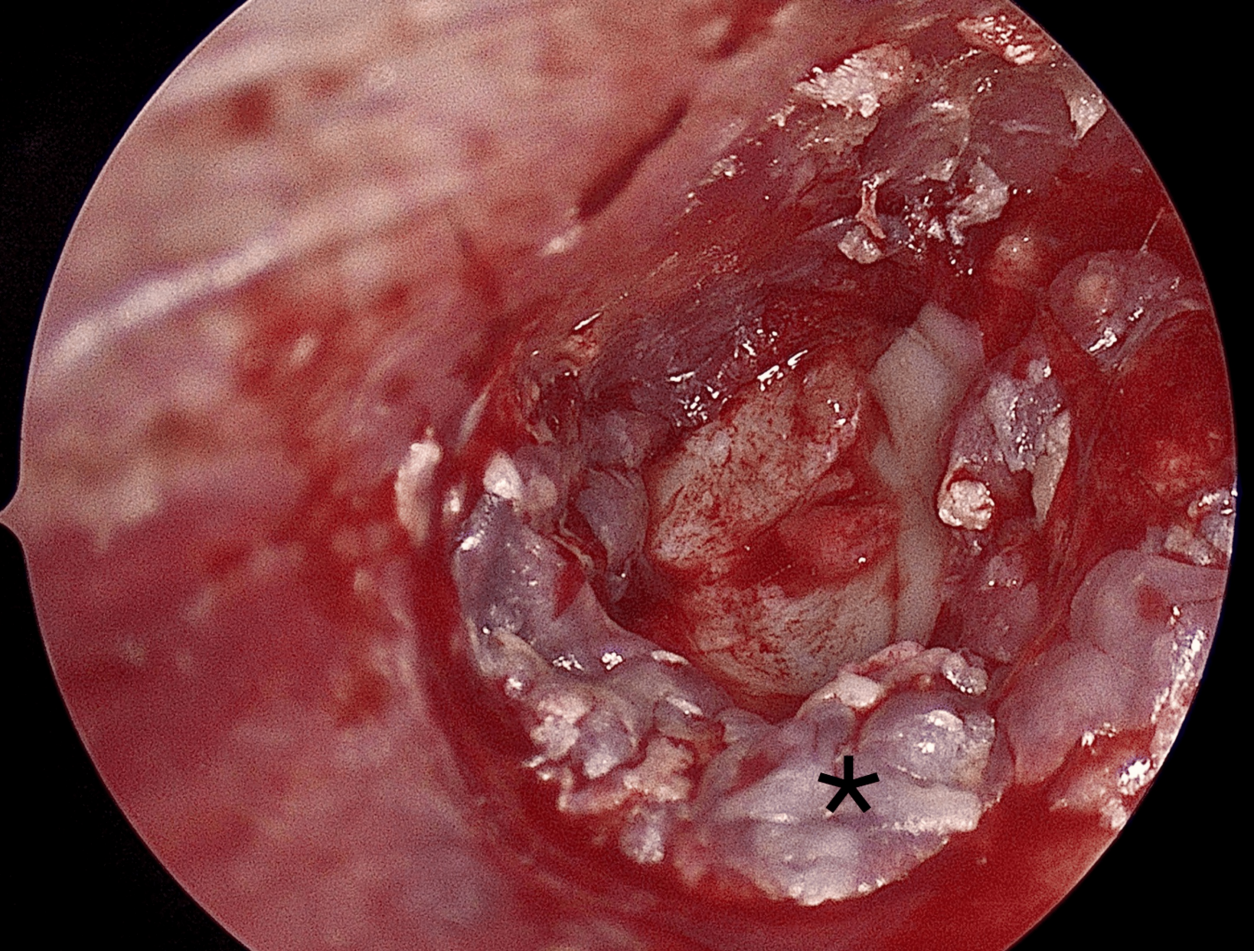
Repositioning the flap (asterisk)

The tympanomeatal flap was carefully repositioned over the graft without folds. Gelfoam was placed in the middle ear for medial support and in the external auditory canal to provide gentle lateral pressure, which helps minimize the risk of graft lateralization (Figure 5).

Postoperative Care

The external auditory canal was filled with Gelfoam and dressed externally. Packing was removed after one week. Patients received oral antibiotics for seven days and topical antibiotic drops for two weeks.

**Figure 6:**
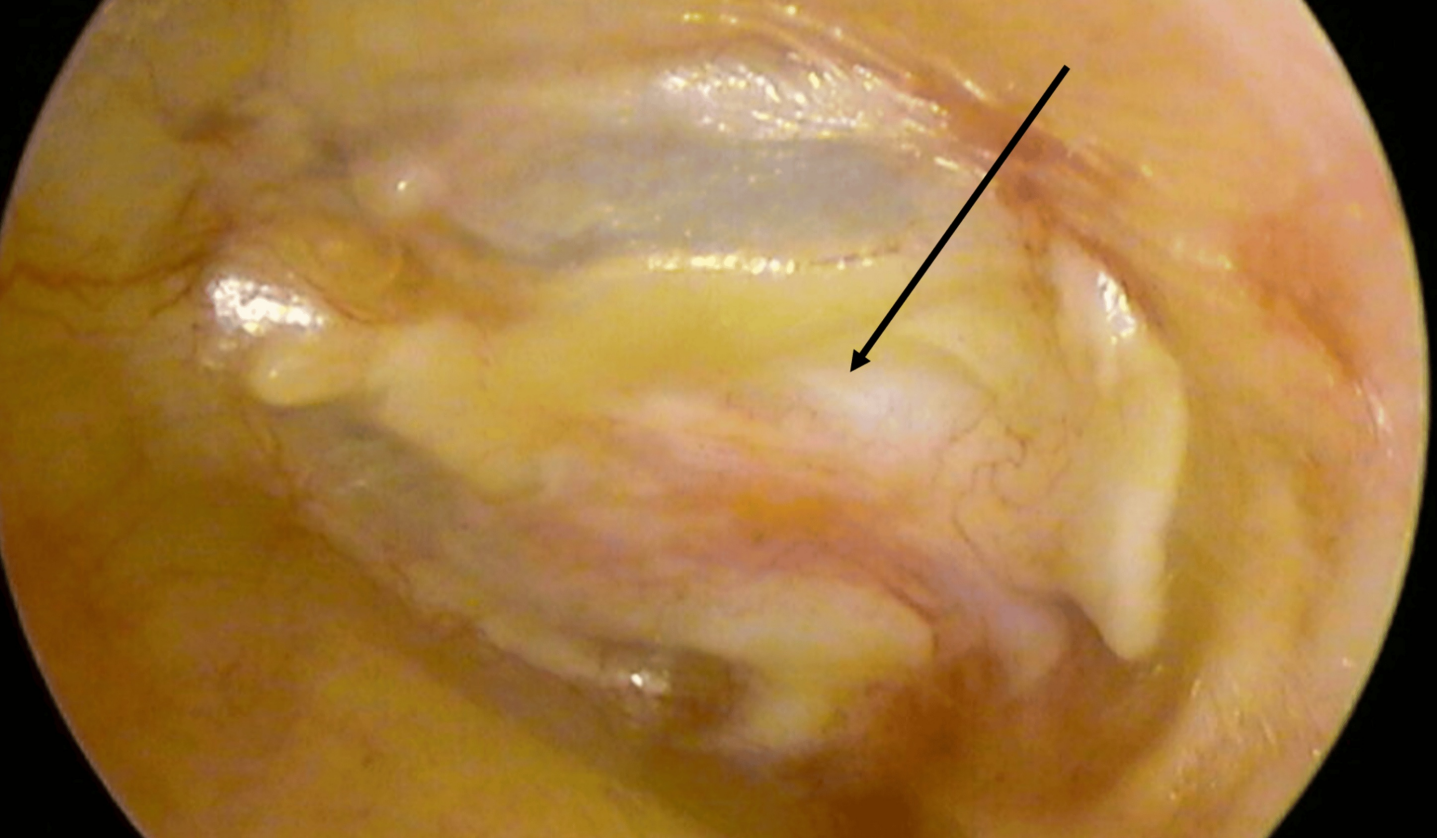
Postoperative endoscopic view at eight months showing complete graft (black arrow) uptake with an intact and well-epithelialized tympanic membrane

On follow-up at one, three, and six months, all five patients achieved complete graft uptake, with full epithelialization by six weeks. No cases of lateralization, anterior blunting, or infection were observed. The postoperative image presented (Figure 6) corresponds to the most recent follow-up at eight months for the illustrated case.

Audiometric evaluation demonstrated a mean preoperative ABG of 28 dB (range 25-32 dB), improving to a mean postoperative ABG of 12 dB (range 10-15 dB), corresponding to a mean closure of 16 dB. Bone conduction thresholds remained stable in all patients, and no complications occurred during follow-up.

Ethical considerations

Formal ethical approval was not required for this retrospective case series, as all procedures were part of standard clinical care and patient identity was not disclosed in any images. Written informed consent for participation and publication was obtained from all patients.

## Discussion

Myringoplasty remains a cornerstone of otologic surgery, with techniques evolving to address anatomical challenges and optimize functional outcomes. The two classical methods, known as underlay and overlay, each present advantages and drawbacks. The underlay technique is straightforward and widely used, but anterior and subtotal perforations remain technically demanding because of visualization difficulties and higher rates of anterior graft failure. The overlay approach provides wide exposure and reliable closure of anterior perforations, but it has historically been associated with longer healing times and complications such as lateralization or blunting.

The introduction of cartilage grafting has significantly improved long-term stability in myringoplasty. Dornhoffer's large series of 1,000 patients demonstrated that cartilage provides durable anatomical success with satisfactory hearing outcomes, even in complex or revision cases [1]. The development of the endoscopic approach further advanced this field. Ayache first described endoscopic cartilaginous myringoplasty via a transcanal approach, showing that endoscopes enhance visualization of the tympanic membrane and facilitate minimally invasive repair [2].

Cartilage grafting has also proven its utility in revision tympanoplasty. Boone et al. reported high closure rates with cartilage in revision cases, even without mastoidectomy [3]. More recently, Bayram et al. compared various graft materials and highlighted the superior reliability of cartilage, particularly in challenging situations [4]. Wang D. and Wang W. also evaluated endoscopic myringoplasty with and without tympanomeatal flap elevation, confirming that both approaches can achieve excellent outcomes when adapted to the surgical context [5]. Finally, Ferlito et al. showed in a long-term retrospective study that cartilage grafts achieve superior graft survival compared with temporalis fascia, with no compromise in functional results [6].

In our series, we employed an endoscopic overlay variant with preservation of the fibrous layer of the tympanic membrane. This technical nuance appears to combine the benefits of the overlay principle with the structural support of the intact fibrous layer, reducing the risk of lateralization while ensuring adequate anterior support. Using tragal cartilage as graft material further reinforced stability. Our results (complete graft uptake, rapid epithelialization, and significant improvement in the ABG) align with the favorable outcomes reported in the literature for endoscopic and cartilage tympanoplasty [1-6].

Although the number of patients was limited, the absence of complications such as lateralization, anterior blunting, or infection supports the feasibility and safety of this variant. Larger prospective studies with longer follow-up will be necessary to confirm its advantages and define its role among established techniques.

## Conclusions

Endoscopic overlay myringoplasty with preservation of the fibrous layer represents a feasible and reliable technical variant for repairing anterior and subtotal tympanic membrane perforations. In our small series, this approach achieved complete graft uptake and significant hearing improvement without complications such as lateralization or anterior blunting. The combination of endoscopic visualization, preservation of the fibrous layer, and cartilage reinforcement may enhance graft stability in challenging cases. Larger studies with longer follow-up are needed to confirm these encouraging results.
